# Compact Wearable Antenna with Metasurface for Millimeter-Wave Radar Applications

**DOI:** 10.3390/ma16072553

**Published:** 2023-03-23

**Authors:** María Elena de Cos Gómez, Humberto Fernández Álvarez, Alicia Flórez Berdasco, Fernando Las-Heras Andrés

**Affiliations:** TSC, Electrical Engineering Department, University of Oviedo, 33203 Gijón, Spain

**Keywords:** metasurface, radar antenna, wearable, millimeter wave, collision avoidance, array antenna, imaging

## Abstract

Three metasurfaces (MTS) are designed to be combined with a series end-fed 1 × 10 array antenna with a modified Dolph-Chebyshev distribution for imaging applications in the millimeter frequency range, 24.05–24.25 GHz. A reduction in secondary lobes and an increase in FTBR can be achieved while preserving gain, radiation efficiency, SLL and size using an MTS–array combination. Moreover, as a result of each single-layer MTS–array combination, operation bandwidth is widened, with gain and radiation efficiency enhancement. The overall devices’ size is 86.8 × 12 × 0.762 mm^3^. The envisioned application is collision avoidance in aid to visually impaired people at a medium-long distance.

## 1. Introduction

In recent years, the literature has been enriched with plenty of research works using metamaterials, especially metasurfaces for various applications, such as RCS reduction, antenna enhancement, wavefront transformation and unconventional waveguiding and scattering [1,2,3,4,5,6,7,8,9,10]. Among them, those aimed at improving antennas [11,12,13,14,15,16,17,18] attract attention due to their interest and commercial applications. On the other hand, there have been numerous advances concerning both, the materials used for fabrication and the fabrication techniques themselves, in terms of the modeling and measurement of metasurfaces [19,20,21,22] and the study of their angular stability [10,11,12,13,14,15,16,17,18,19,20,21,22,23], as well as their influence on the operation of the antennas with which they are combined.

Moreover, the use of increasingly higher frequencies, such as millimeter-wave frequencies, is spreading, due to the large bandwidths that can be allocated, with corresponding advantages in throughput for 5G and other applications, such as radar.

The aim of this work is to investigate whether it is possible to improve all the properties of an antenna at once, both its impedance-matching bandwidth and its radiation properties, without increasing its overall size, using metasurface-based techniques. This approach contrasts with the traditional methods carried out so far, in which either artificial magnetic conductors (AMCs) are used under the antenna at some distance (or replacing the ground plane of the antenna) increasing the thickness, or EBGs with numerous cells surrounding the initial antenna (increasing its size), or employing frequency selective surfaces, polarizers or partially reflecting surfaces over the antenna at some distance to modify its radiation pattern, always increasing the thickness. All of the above methods increase the cost and complicate the antenna manufacturing process. In addition, these techniques improve only some of the antenna parameters, but not all at once (or considerably improve some at the expense of certain deterioration of others). Other recent works including [24] focus on either trying to reduce the size of an array of microstrip patches with corporate (parallel) feeding, improving some of its radiation parameters, but slightly worsening others and keeping the bandwidth, or slightly improving all the radiation parameters but not the bandwidth, while preserving size, based on a reduction in the coupling between the array patches by arranging a wall of unit cells between them and the suppression of the surface waves, with more than one row of unit cells in the front part.

Another important aspect to highlight is that most of the works that involve the combination of an antenna with a metasurface, usually derive in a modification of some of the dimensions of the metallization of the antenna to achieve that the antenna–metasurface assembly works conveniently, even when a certain distance is left between both. Therefore, the comparison of the antenna alone with the metasurface–antenna combination loses rigor, since it is not the same antenna. Consequently, the conclusions drawn from the point of view of the usefulness of the metasurfaces or the physical phenomena involved are more than debatable, even if an operational device is achieved.

Concerning the state-of-the-art on wearable antennas with metasurfaces, there have been significant advances for biomedical applications [25], as well as those related to WLAN [26], WBAN [27] and wireless power transfer, either in microwave or millimeter-wave frequencies. However, some of these contributions involve metallic vias [25], which make the fabrication more complex and expensive, as well as not being best suited to be wearable or long-lasting. Most of the works require the combination of two-dimensional composite right- to left-handed transmission line (2D CRLH-TL) and AMC metasurfaces [25,26,27,28,29,30,31], which have to be placed below the initial antenna to isolate the antenna from the body, which, as mentioned above, implies increasing its thickness and sometimes also its size. Sometimes, the AMC-backed antenna provides bandwidth broadening [30], which can also be achieved by an L-probe-fed metasurface antenna [31]. Whereas other authors propose bandwidth enlargement combining the antennas with EBG metasurfaces [32], which can also be used to suppress surface waves and isolate the antenna from the body [33], as well as for improving the front-to-back ratio (FTBR) [34]. Moreover, involvement of both EBG and AMC metasurfaces is required to obtain beam-switching functionality and reduce the backward radiation [35]. Finally, Wang et al. [36] proposed the use of an AMC arranged on the side of a monopole antenna to achieve a directional button-type antenna for power transmission over the human arm, avoiding its undesired effect on propagation. Once again, when the EBG is arranged on the back of the antenna it increases the resulting thickness, whereas when the unit cells surround the initial antenna the size increases.

The aim of this work is to design an antenna with improved performance for a specific radar application in the millimeter frequency range, specifically in the ISM band from 24.05 GHz to 24.25 GHz, due to the suitability of such frequencies for high-resolution detection in foggy, smoky and dusty environments. It is intended for use in a collision-avoidance-based system to assist visually impaired people and therefore must be compact and lightweight, efficient and radiate as little as possible in the direction of the human body (which translates into a high front-to-back ratio). The antenna has a major impact on radar performance. A compromise solution between range and coverage area has to be adopted. In addition, it must have an appropriate directivity to be able to detect obstacles at the intended distance and it also influences the angle determination of the target.

Regarding the design and analysis of the metasurface to be combined with the antenna, it is not only intended to be operative in the frequency band of interest (24.05 GHz to 24.25 GHz), but also to analyze its complete angular stability, that is, taking into account both the variation of the electric field polarization angle, Phi, from 0 to 90 degrees, and the angle of oblique incidence, Theta, from 0 to 60 degrees, both for TE- and TM-polarized incident plane waves. It should be noted that most angular stability studies only consider the Phi = 0 case, leaving relevant cases unanalyzed, which is not convenient when intending to combine metasurface with antennas. The angular stability in TM can be improved by reducing the thickness of the metasurface dielectric, although this reduces the operating bandwidth, so a compromise must be found between both parameters, as well as considering the availability of commercial substrates.

The paper is organized as follows: first, Section 2 “Materials and design methods” briefly describes the array antenna, which is the reference device, and more in depth the design of the three metasurfaces that will be combined with this antenna, analyzing their resonance frequency, reflectivity, surface impedance and angular stability. This section also includes the results obtained in simulation, in terms of impedance-matching bandwidth and radiation properties, both for the antenna alone and for the antenna combined with the metasurfaces under study and also with a metallic parasite of the same size. Then, Section 3 “Experimental results” shows the prototypes manufactured corresponding to the designs conducted in the previous section, as well as the results obtained from their measurements in terms of impedance matching and radiation pattern in an anechoic chamber. Next, Section 4 “Discussion” aims at explaining the most significant findings of this study and discussing how they can be interpreted and their implications. Finally, some relevant conclusions are drawn in Section 5.

## 2. Materials and Design Methods

**Figure 1:**
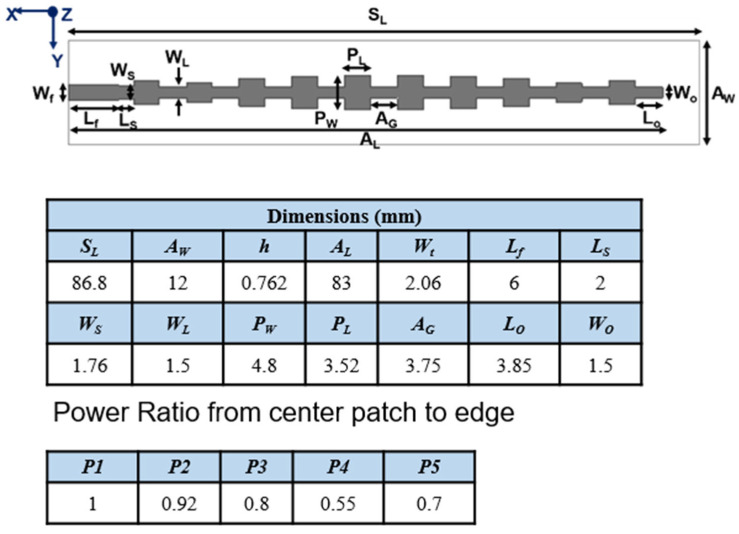
Antenna geometry and dimensions.

A recently published series end-fed 1 × 10 array antenna on polypropylene [37] will be used as a starting point for this study, but slightly reducing the width of the ground plane, which makes it even more compact and wearable. In the present work, RO3003 (ε_r_ = 3.0 and tan δ = 0.0013) [38] is used as the dielectric substrate for both the antenna and the metasurfaces, as the aim is to explore the potential improvements that metasurfaces bring to the starting antenna, without further increasing the complexity by using unconventional dielectrics that add even more challenges in fabrication, given the small dimensions for high-frequency operation. The aforementioned antenna involves a modified Dolph-Chebyshev distribution for improved beam width. Figure 1 shows the antenna geometry, its dimensions and the patch width ratio for optimized performance, not only in terms of radiation properties but also concerning impedance-matching bandwidth.

From the S_11_(dB) results obtained in simulation for the optimized array antenna design, the operation bandwidth with suitable impedance matching is 23.98–24.42 GHz, being optimal in the intended 24.05 GHz to 24.25 GHz ISM band. The radiation characteristics of the antenna obtained in simulation, in terms of peak realized gain (G), peak directivity (D), radiation efficiency (η) and front-to-back ratio (FTBR), are indicated in Table 1 for the center and end frequencies of the target band.

In addition to the high G (>14.0 dBi) and D (>14.5 dB) levels achieved in the whole band, it is noteworthy the levels of both the high radiation efficiency (>90%) and the FTBR (>20 dB), which are critical in a wearable application. The side-lobe level (SLL) and the half-power beam width (HPBW) at the center frequency of the band (24.15 GHz) are SLL_Phi0_^°^ = −16 dB, HPBW_Phi0_^°^ = 12°, SLL_Phi90_^°^ = −28 dB and HPBW_Phi90_^°^ = 64°. All of these results are suitable for the envisioned collision-avoidance application. However, while this antenna can be said to meet the requirements in the commercial 24 GHz band, and there is scarce margin for improvement within it (perhaps try to increase somewhat radiating efficiency and FTBR and reduce secondary lobes), it is well known that if the operational bandwidth of the antenna is widened, this results in a higher resolution and improvement in the detection of obstacles, which is the main objective of this application. Therefore, by using a wider bandwidth radar transceiver, end-user performance would be improved. Thus, if it is possible to widen the antenna’s operating frequency band while preserving (or even improving) the characteristics of the radiation pattern, without increasing the size and neither complicating nor making manufacturing more expensive, it would be a significant achievement for the aim pursued.

**Figure 2:**
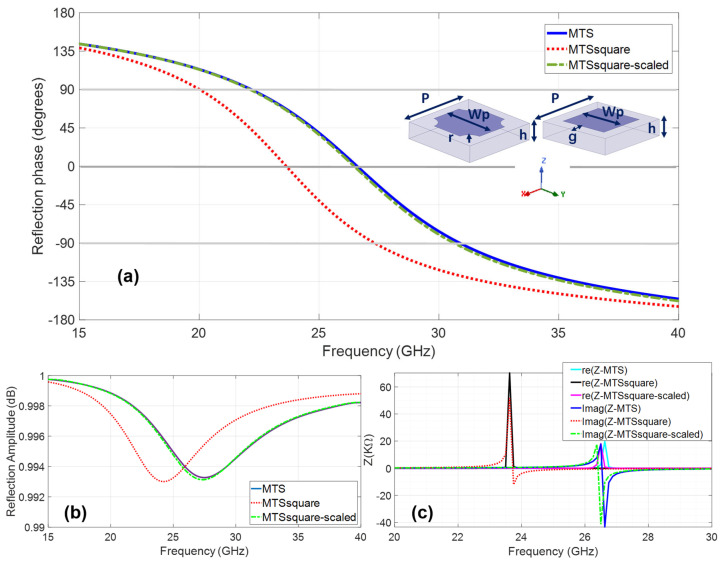
Metasurfaces: (**a**) reflection coefficient phase and unit cells dimensions, (**b**) reflection coefficient amplitude and (**c**) surface impedance.

Three high-impedance metasurfaces (MTS) [19] are designed for operation in the intended ISM 24.05 GHz to 24.25 GHz radar band to be arranged around the array antenna, pursuing a suitable antenna for wearable medium-long distance collision-avoidance radar [39,40]. Figure 2 shows the reflection coefficient phase and amplitude, the surface impedance and the unit cell dimensions for the metasurfaces, all of them with the same periodicity P = 3.6 mm and thickness h = 0.762 mm. The differences lie in the geometry of the metallization and/or in the size of the metallization (Wp) and the size of the gap (g). There is one design with square metallization size Wp = 3.6 mm, with rounded corner cuts with radius r = 0.57 mm and gap g = 0.5 mm, henceforth referred to as MTS. In addition, there are two metasurface designs with square unit cell metallization, one with metallization size Wp = 2.6 mm and gap g = 0.5 mm referred to as MTSsquare and other with Wp = 2.37 mm and g = 0.615 mm hereafter referred to as MTSsquare-scaled.

It can be observed that under normal incidence, MTSsquare resonates at 23.66 GHz, very close to the antenna’s operating frequency band, with in-phase operation from 20.07 GHz to 27.32 GHz, whereas MTS and MTSsquare-scaled resonate at a higher frequency, 26.58 GHz, with in-phase reflection operation from 20.07 GHz to 30.9 GHz, therefore still exhibiting in-phase reflection and increasing surface impedance in the antenna’s operating band. The three metasurfaces can be considered high-impedance surfaces (HIS) in view of their surface impedance shown in Figure 2c.

**Figure 3:**
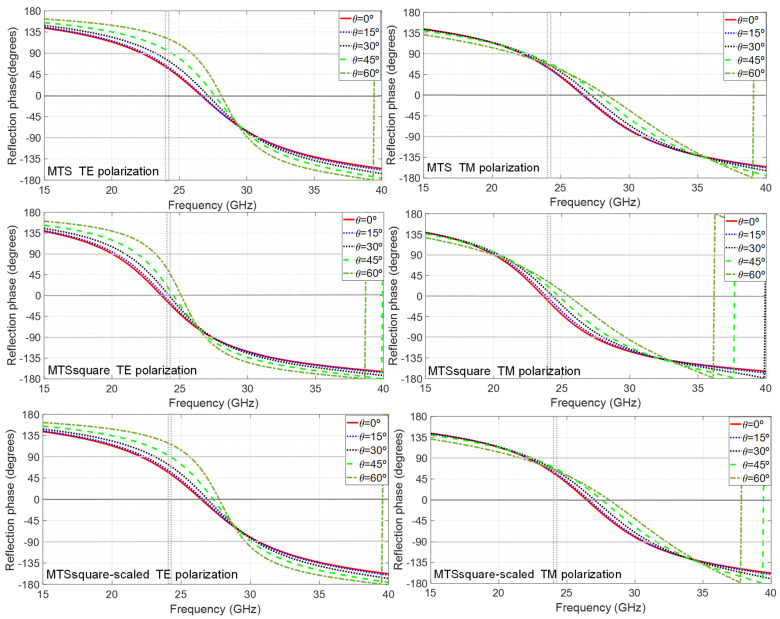
Simulation results of metasurfaces’ reflection coefficient phase for TE and TM polarization under different incidence angles θ = 0°, 15°, 30°, 45° and 60° corresponding to a φ = 45° polarization angle of the incident field.

The angular stability of the metasurfaces has been analyzed. The incidence angle θ is varied from 0° to 60° in steps of 15° for each polarization angle of the incident electric field φ which is, in turn, varied from 0° to 90° in steps of 15°. Figure 3 shows the results for the reflection coefficient phase for φ = 45° which has turned out to be the worst case, exhibiting lower angular stability. The three metasurfaces can be considered very stable in the bandwidth of interest, especially for TE polarization, since they keep yielding in-phase reflection up to at least an oblique incidence angle θ = 45°. Comparing the metasurfaces with each other, the MTSsquare is completely stable up to at least θ = 60°, so it could be considered the most stable of the three designed.

**Figure 4:**
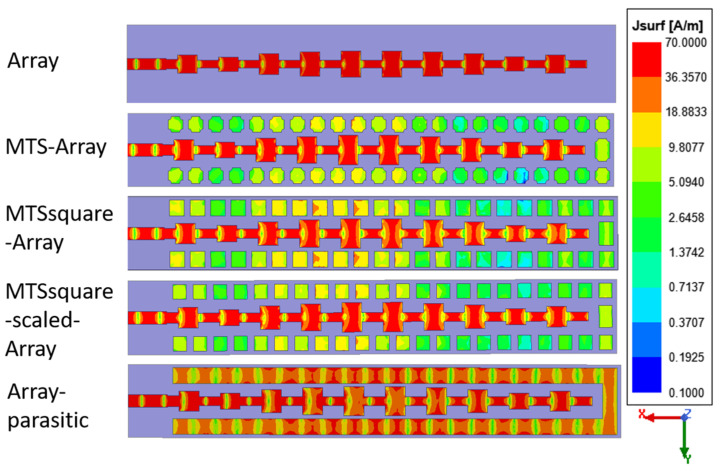
Surface current distribution at 24.15 GHz for the antennas under analysis.

The metasurfaces are arranged around the antenna, as shown in Figure 4, also considering the case of a metallic parasite of the same size placed at the same distance from the antenna, since the placement of parasites is a method used to increase the bandwidth and/or increase the gain.

The current distribution at the center frequency of operation (24.15 GHz) for the array antenna alone and combined with the metasurfaces and the metallic parasite is shown in Figure 4. As it could be expected, for the array antenna, each and every one of the patches exhibit a similar distribution, with a high current level at the center and decreasing to the edges in the X (feeding) direction, since all of them are contributing to the antenna operation. Such current distribution is preserved when the metasurfaces are arranged around the array. However, this does not happen in the case of arranging the metallic parasite, but rather the current distribution is perturbed, although it is located at the same distance and occupies the same area as the metasurfaces. Therefore, even though a small number of unit cells are being used in the metasurfaces and, more specifically, they are periodic in only one direction, their behavior is not that of a parasite. Their resonant behavior, in-phase reflection band and high surface impedance play a role when combined with the antenna.

**Figure 5:**
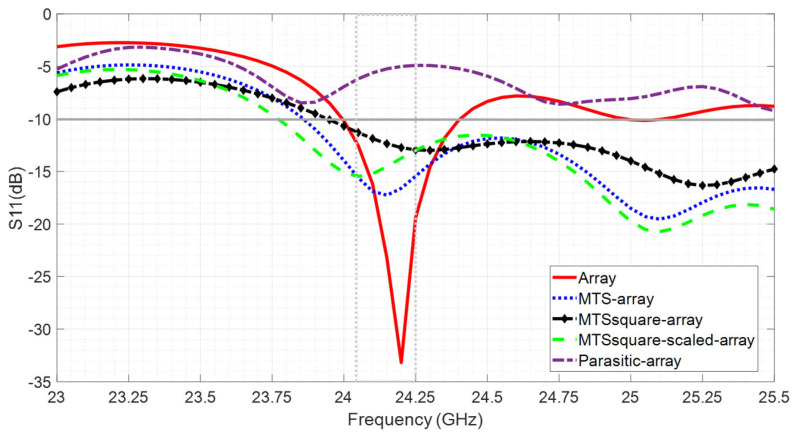
Simulation results for the reflection coefficient, S_11_(dB), for the antennas under analysis.

Analyzing the effect of the aforementioned combinations with regard to the frequency band with proper impedance matching, it can be seen in Figure 5 that the three metasurfaces considerably widen this band, whereas the metallic parasitic mismatches the antenna and renders it inoperative in the required band.

As for the radiation properties within the 24.05–24.25 GHz frequency band and given in Table 1, it can be observed that the radiation efficiency slightly improves, especially for metasurfaces resonating at a frequency higher than the operating one of the antenna (MTS and MTSsquare-scaled), always preserving G > 14.0 dBi, D > 14.3 dB and the values of SLL and HPBW for Phi = 0°. Moreover, the secondary lobes are reduced. For Phi = 90°, the main lobe slightly narrows and the SLL decreases for MTS and MTSsquare; however, given the shape of the radiation pattern, this lobe is on the back-side for the Phi = 90° pattern cut, so it is less relevant. It can even be said that there is an improvement in the FTBR, when the criteria of considering the backward radiation at an angle located 180° far from that of maximum forward radiation (as the commercial software used in the simulations does) are applied. However, for the desired application, it is more appropriate to consider the maximum forward and rearward directivity for the FTBR calculation, regardless of the exact rear angle, as stated in [41], since it is intended that the antenna radiates as little as possible toward the user. In this sense, the FTBR of the array is 17 dB and is preserved when the metasurfaces are added. The cross-polar (XP) level slightly increases with the arrangement of the metasurfaces but still keeps at a suitable level for the pursued application.

The most significant advantage comes from the fact that the highlighted increase in bandwidth is accompanied by an improvement in gain and radiation efficiency, and preservation of the radiation pattern shape and the FTBR (which is improved in some cases).

**Figure 6:**
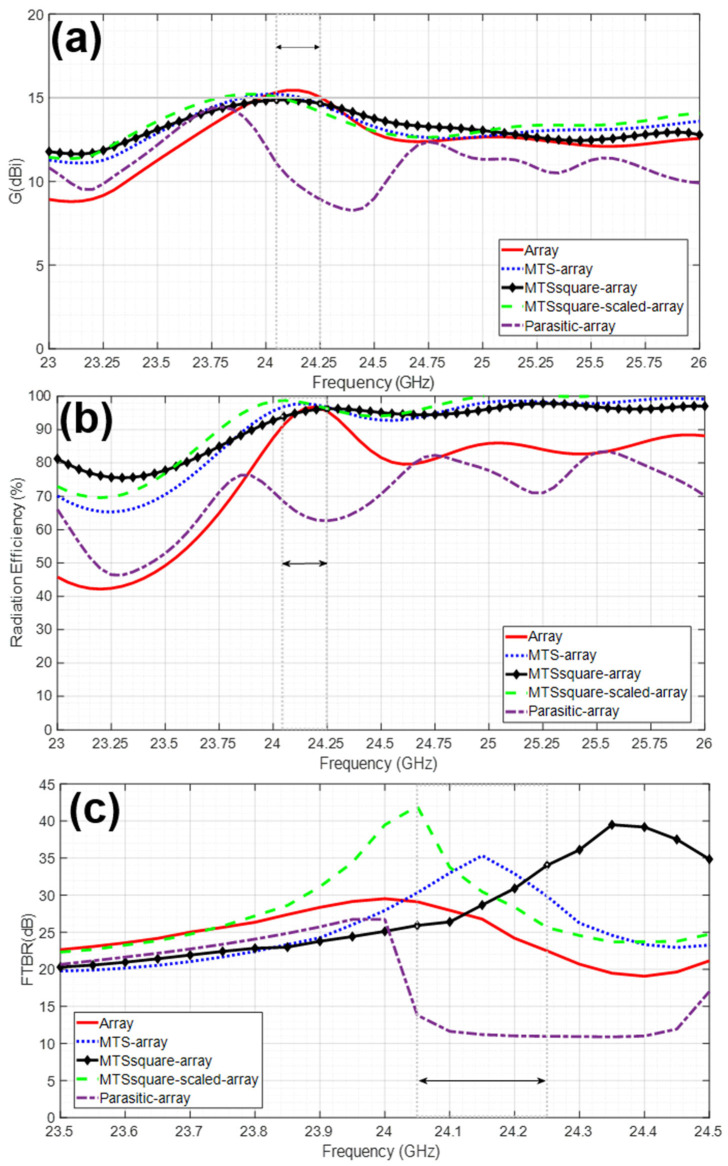
Simulation results for (**a**) gain (dBi), (**b**) radiation efficiency (%) and (**c**) front-to-back ratio FTBR (dB) for the antennas under analysis.

**Figure 7:**
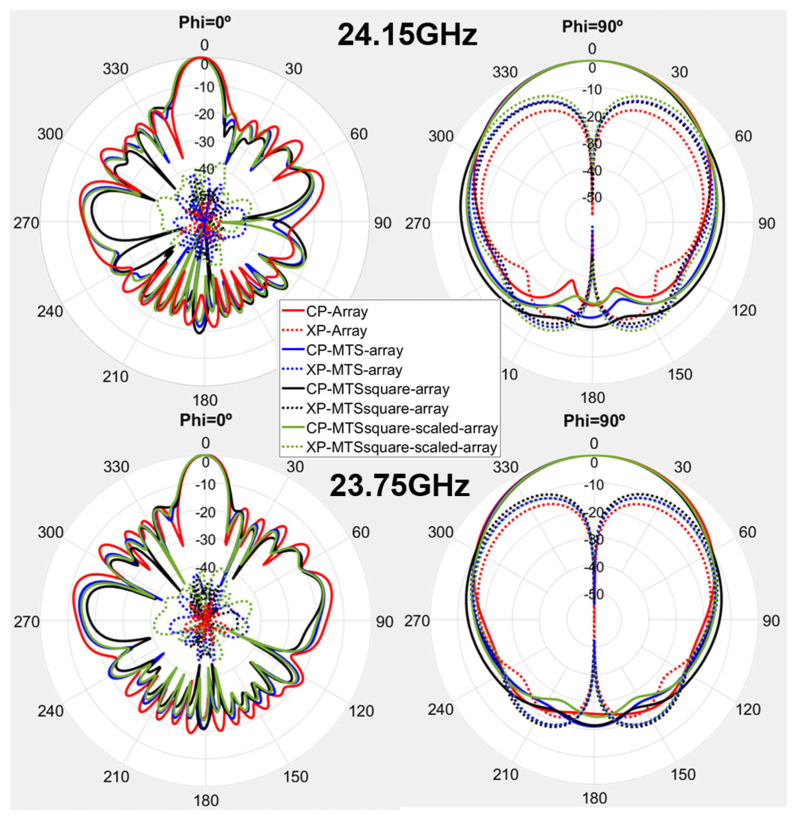
Radiation pattern cuts for Phi = 0° and Phi = 90° at 24.15 GHz and 23.75 GHz for the antennas under analysis.

As an advantageous example of the inclusion of a metasurface, it can be observed in Figure 5 that the MTSsquare-scaled–array combination is well matched at 23.75 GHz (whereas the array is not) and provides a gain of 15 dBi and a radiation efficiency of 90% compared to 13 dBi and 65% provided by the array and without degrading FTBR, SLL and HPBW (see Figure 6 and Figure 7).

**Table 1:** Radiation properties for the array antenna alone and combined with metasurfaces and parasite.

	Freq (GHz)	G (dBi)	D (dB)	η (%)	FTBR (dB)	SLL (dB) φ = 0°	HPBW (°) φ = 0°	SLL (dB) φ = 90°	HPBW (°) φ = 90°
**Array**	24.05	15.1	15.4	93	27				
	24.15	15.2	15.3	97	27	−16	12	−28	64
	24.25	14.7	14.9	96	24				
**MTS–Array**	24.05	15.2	15.4	97	30				
	24.15	15.0	15.1	98	35	−16	12	−25	58
	24.25	14.6	14.8	97	30				
**MTSsquare–Array**	24.05	14.9	15.1	94	26				
	24.15	14.8	15.0	96	29	−15	12	−21	55
	24.25	14.7	14.8	96	34				
**MTSsquare-scaled–Array**	24.05	15.0	15.1	99	42				
	24.15	14.7	14.8	98	30	−15	12	−30	57
	24.25	14.2	14.3	96	26				
**Array–Parasite**	24.05	11	13	68	14				
	24.15	10	12	64	11	−8	8	−34	40
	24.25	9	11	63	11				

## 3. Experimental Results

**Figure 8:**
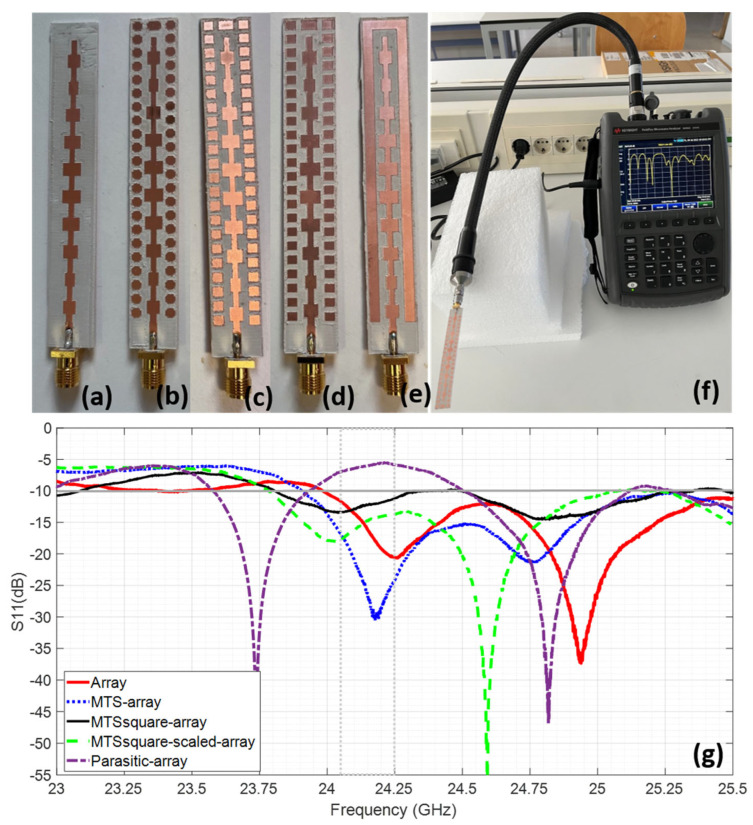
Fabricated prototypes of the antennas under analysis: (**a**) array, (**b**) MTS–array, (**c**) MTSsquare–array, (**d**) MTSsquare-scaled–array and (**e**) array–parasite; (**f**) measurement set-up for the reflection coefficient, S_11_(dB) and (**g**) measurement results for the reflection coefficient, S_11_(dB), of the fabricated antennas.

Conventional micromachining is used to fabricate the prototypes, and an SMA connector operating up to 26 GHz is soldered by hand to feed them (see Figure 8a–e which includes the resulting prototypes).

### 3.1. Impedance Matching

The measured reflection coefficient for the fabricated antennas is depicted in Figure 8. It can be observed that all the prototypes exhibit suitable impedance-matching levels at the target ISM radar frequency band, from 24.05 GHz to 24.5 GHz. Nonetheless, fairly good agreement with the simulation results is achieved, allowing to observe the expected widening of the frequency band. It has to be taken into account that the connector was not included in the simulation and it is soldered by hand, so that it causes a slight disturbance in terms of slightly shifting and/or broadening the frequency band.

### 3.2. Radiation Properties

**Figure 9:**
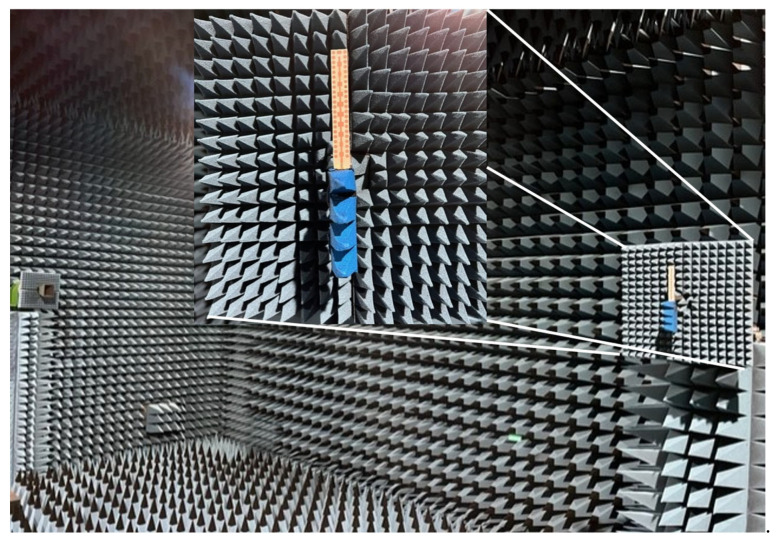
Measurement set-up in an anechoic chamber for radiation properties with zoomed detail of an antenna under test (AUT).

**Figure 10:**
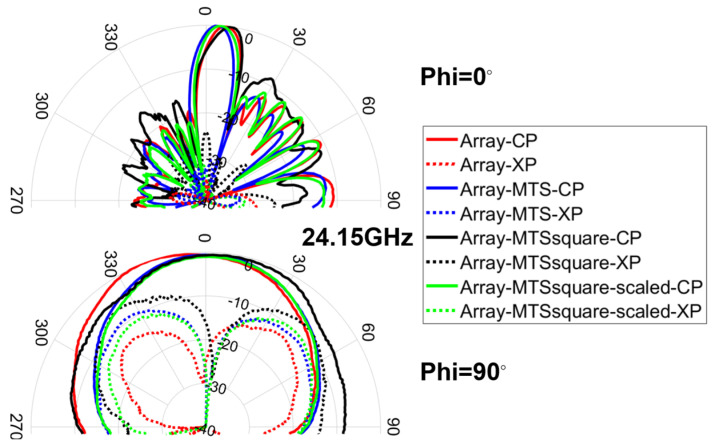
Measurement results for the co-polar (CP) and cross-polar (XP) radiation pattern components of the antennas under analysis for Phi = 0° and Phi = 90° cuts at 24.15 GHz.

The radiation pattern cuts for Phi = 0° and Phi = 90° at the center frequency of the band (24.15 GHz) were measured in an anechoic chamber (see Figure 9), and the results are depicted in Figure 10 for both co-polar (CP) and cross-polar (XP) components.

Fairly good agreement is achieved between simulation and measurement results, especially for the CP components. The resulting HPBW level is almost identical to the one obtained in simulation for both Phi = 0° and Phi = 90° cuts, whereas the SLL slightly worsens in measurement for Phi = 0°. All the antennas under analysis exhibit an asymmetry in the Phi = 0° plane due to the feeding method, which is already observed in the simulation results. This makes Phi = 0° pattern cut more sensitive to everything that is actually located in the feeding part of the antenna. In the measurement results, both the tilt and the regrowth of the side lobes observable on one side of said pattern cut are attributable to the effect of the connector and especially of the bend (right-angled adapter or 90-degree bend) between the cable and the connector, which are not considered in the simulation. Both the connector and the bend enlarge the effective length of the antenna and modify the current distribution at its input. In addition, the movement of the cable when rotating the positioner to measure said pattern cut further contributes to modify the current distribution and exerts mechanical tension pulling the antenna, which may cause slight differences in positioning between antennas.

Again, it should be noted that the connector was soldered by hand on each antenna. Both the cable and the connector can severely perturb the current distribution on the small antenna and be also responsible for the observed XP levels in measurement.

The gain transfer method, which involves inter-comparison of the array antenna prototype (antenna under test (AUT)) with a Flann Microwave Standard horn 20240-25 (probe antenna of known characteristics), was used to conduct the measurements of the peak realized gain. It has to be taken into account that, in addition to the aforementioned effects of the cable and the connector, the gain measurement can be disturbed by other effects [42]: multipath and reflections due to the fixing PLA structure and other set-up elements, misalignment of AUT-probe and impedance mismatch of antennas. Thus, some reduction in the gain level is observed compared to the simulation results but being consistent with them and attributable to the aforementioned facts (see Table 2).

## 4. Discussion

The most significant finding of this work is that the operating bandwidth of an antenna can be widened, without degrading or even improving its radiation characteristics, without the need to increase its size, using metasurfaces with a reduced number of unit cells.

**Figure 11:**
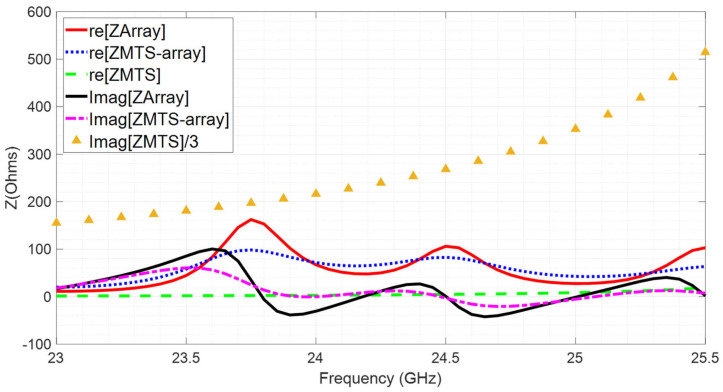
Impedance of the array, the MTS–array combination and the MTS versus frequency.

To explain the broadening of the impedance-matching bandwidth, the impedances of the antenna and the metasurfaces have been analyzed. The reactance of the antenna alone (the imaginary part of the array impedance represented in Figure 11) is capacitive in the 24.05–24.25 GHz band, while the metasurfaces exhibit a high inductive reactance in that band. The arrangement of the metasurfaces around the antenna can be modeled in circuit terms as the impedance of the antenna connected in parallel with that of the metasurface with a series coupling capacitor between them, due to the gap distance. As a result of the combination of the antenna and the metasurface, a reduction in reactance is obtained, due to a compensation effect of the capacitive and inductive reactances, even when a reduced number of unit cells is being used in the metasurface. Figure 11 shows the impedances of the array alone, of the MTS (the imaginary part of has been divided by 3 for better visualization) and of the MTS-array combination. The aforementioned reactance compensation can be observed, coinciding with the frequency band in which good impedance matching is achieved.

**Figure 12:**
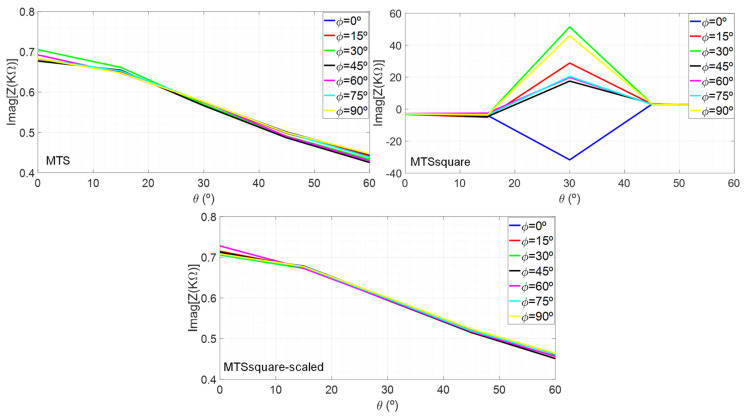
Imaginary part of the surface impedance at 24.125 GHz for the antennas under analysis.

The differences between the designed metasurfaces can be explained in terms of their angular stability, especially in terms of their surface impedance. The variation of the imaginary part of the surface impedance has been analyzed in terms of the oblique incidence angle, θ, for different field polarization angles, φ. Figure 12 shows the results obtained at the frequency of 24.125 GHz. It can be seen that the variation for MTS and MTSsquare-scaled is much smaller than for MTSsquare, since they are operating further away from resonance. In addition, it was observed that when increasing the frequency in the 24.05–24.25 GHz band, the range of variation is similar, the trace only shifts upwards, preserving the shape. However, for MTSsquare, the values change significantly for small frequency variations, because they are closer to resonance. This explains why MTS and MTSsquare-scaled provide a wider bandwidth and better impedance-matching values within it. In addition, MTSsquare provides worse performance in terms of G, D, η, SLL and main beam narrowing for Phi = 90° pattern cut, than the other metasurfaces. In the current distribution of Figure 4, it can be observed that, although it does not degrade those of the array, they differ more than in the case of the other metasurfaces, and the value of the currents in the unit cells close to the center of the array is higher, leading to a greater modification in the radiation pattern.

Tangential components of the electric field, Ex and Ey, in a plane located a short distance above the antenna (field at the antenna aperture) are obtained and analyzed for further insight into the antennas under analysis.

**Figure 13:**
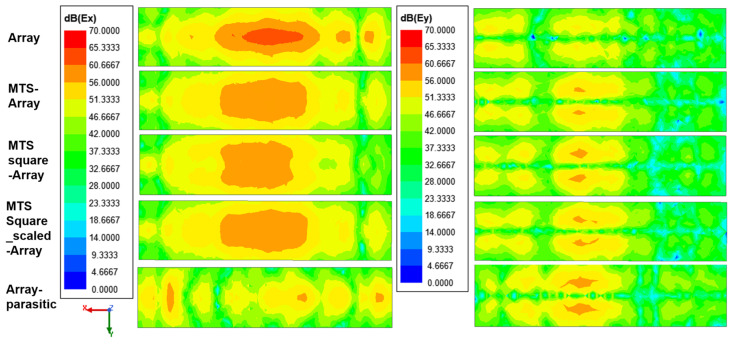
Tangential components of the electric field, Ex and Ey, at 24.15 GHz for the antennas under analysis, in a plane located a short distance above the antenna.

In view of the results shown in Figure 13, the fields at the antenna aperture are more similar to each other for the MTS–array and MTSsquare-scaled–array combinations, especially the Ex component, as would be expected, since these metasurfaces exhibit nearly identical behavior. This is also in line with the distribution of currents in Figure 4, which shows similar levels in both the array and the metasurface cells for those combinations.

The Ey level is slightly higher for the MTSsquare-scaled–array, which agrees with a higher value of the cross-polar component observed in the radiation pattern of Figure 7. In all cases, the metasurfaces have the effect of broadening the field distribution in the aperture and of raising the value of the Ey component, which, in addition to increasing the level of the cross-polar, narrows the main lobe for Phi = 90, which is especially notable in the case of the MTS-square–array (see Table 1). In the case of the metallic parasite, the distribution of the field in the antenna aperture has nothing to do with that of the initial array, which agrees with the disturbance in the current distribution observed in Figure 4 and the consequent degradation of the radiation properties.

## 5. Comparison with Other Millimeter-Wave Antennas at 24 GHz Radar Band

To assess the relevance of the antennas presented in this work, a comparison with other state-of-the-art antennas operating in the 24 GHz radar band is presented in terms of size, bandwidth and radiation properties (see Table 3). There are hardly any portable antennas in the 24 GHz band and those that exist are either for applications whose requirements differ greatly from those of the intended application and/or use textile substrates (such as felt, denim) or plastics (PET, PVC, PP, etc.), thus with relative permittivity values between 1.2 and 2.3. Most of the antennas in the target band in the literature are for vehicle anti-collision radars or rectennas for energy harvesting and for wireless power transfer to wearable sensors, as well as biomedical applications. The requirements of those antennas for in-body medical applications and the rectennas are very different from the ones demanded in collision-avoidance systems, and so the latter ones are those with requirements closer to the antennas presented here in terms of exigence. For a fair comparison, antennas on substrates with relative dielectric permittivity values very close to that of the antennas presented in this work (i.e., ε_r_ =3) should be considered. Otherwise, the values of impedance-matching bandwidth and antenna dimensions will not be comparable. Neither would be those radiation parameters affected by potential surface wave propagation, which increases for high ε_r_ (and even more so the thicker the substrate). Therefore, the range will be limited to ε_r_ values between 2.9 and 3.66.

It is also noteworthy that most of the works in the literature do not provide the results of radiation efficiency and FTBR.

Regarding the antennas on substrates with identical ε_r_ values or as close as possible to that of the antennas presented in this work [24,43,44,45,46], all of them provide G results much lower than the ones required in the target application. Although [43] shows a considerable bandwidth and is quite compact, it is on paper, which makes it unsuitable in contact with sweat or humidity and not very robust. Moreover, its radiation efficiency is one-third of that achieved by the designs proposed in this work. It also stands out [24] for the G and BW that it provides considering its small size, although its performance is outmatched by the proposed antennas. The antenna designs corresponding to ε_r_ values, which are slightly higher than 3 and lower than 3.6, [40,47,48,49,50], provide results of G lower than 14 dBi and with BW similar to that shown by the basic antenna of the present work [47,48,50], and in all cases lower than that provided by the antennas with metasurfaces proposed in this work. It can be highlighted [48] with G = 13 dBi, which could be compared with the basic array proposed here, although [48] with lower BW and G, and taking up more area despite using a higher ε_r_. Finally, Refs. [51,52,53], which are for applications more directly related to the one pursued, exhibit higher G but also lower BW than the metasurface proposals, and all occupy larger area even with higher ε_r_, making them less advantageous in terms of compactness. It should be clarified that [51] uses a 12 × 1 × 8 array and so provides such a high gain. If several arrays like the ones presented here are put in parallel, such high values of gain can be achieved.

**Table 3:** Millimeter-wave antennas operating at 24 GHz radar band.

Ref.	Size (mm^3^)	ε_r_	BW (%)	G (dBi)	η (%)	FTBR (dB)	SLL (dB) φ = 0°	HPBW (°) φ = 0°	SLL (dB) φ = 90°	HPBW (°) φ = 90°
[43]	20 × 20 × 0.68	2.9	8.3	7.4	35	-	-	54	−25	48
[24]	26.7 × 20.4 × 0.762	3.0	7.1	9.2	100	17	−16	54	−5	32
[44]	>76 × >76 × 0.13	3.0	3.8	4.8	-	-	-	-	-	-
[45]	17.2 × 17.2 × 0.18	3.0	2.5	5.0	-	-	-	60	-	60
[46]	6.8 × 6.8 × 0.26	3.0	2	5.44	-					
[47]	36.5 × 53 × 0.1	3.35	0.8	5.81		-	-	65	−7	20
[48]	67 × 16.5 × 0.254	3.48	1.5	13	-	-	−26	17.5	-	-
[40]	29 × 21 × 0.254	3.48	2.1	10.4	-	-	-	58	-	47
[49]	23 × 15 × 0.254	3.48	6.6	4.24	-	9.23	-	-	-	-
[50]	90 × 25 × 0.203	3.55	1.6	11				82		17
[51]	135 × 58.7 × 0.254	3.6	2.7	24.2	-	22	−23	12.5	−23	5.6
[52]	50 × 59 × 0.254	3.66	1.0	20.6	-	-	−18	19.2	−19	12.2
[53]	50 × 50 × 0.754	3.66	6.3	20.9	-	-	−21	15	−22	28
Array *	86.8 × 12 × 0.762	3.0	1.8	15.2	97	27	−16	12	−28	64
MTS-Array *	86.8 × 12 × 0.762	3.0	>8.5	15.0	98	35	−16	12	−25	58
MTSsquare-Array *	86.8 × 12 × 0.762	3.0	>8.5	14.8	96	29	−15	12	−21	55
MTSsquare-scaled-Array *	86.8 × 12 × 0.762	3.0	>8.5	14.7	98	30	−15	12	−30	57

* This work at 24.15 GHz.

Provided that millimeter-wave energy penetrates the stratum corneum easily but is rapidly absorbed within the deeper epidermis and dermis and does not propagate further into the body [54] and that the presented antennas are metal-backed, their performance will hardly be affected by the human body (as would be the case for antennas without a ground plane or at lower frequencies). Furthermore, at 24.15 GHz, the penetration depth is 1.0888 mm [55], which means that the energy does not get through the 2 mm or 1.5 mm skin layer thickness of the tissue models on the chest or on arm [55,56]. Although the radiation efficiency will drop slightly (less than 10%), it will remain at more than acceptable values for the intended application (higher than 84%). The bandwidth would not be degraded, since the human body in this band acts almost as an even larger ground plane. Moreover, the presented antennas could not only be arranged on the chest or a bracelet on arm, but also, for example, be placed in the frontal frame of glasses, thus, without direct contact to the human body.

## 6. Conclusions

It has been proven that the operating bandwidth of an antenna can be widened, without degrading or even improving its radiation characteristics, while maintaining both its size as well as its ease and cost of manufacturing, using metasurfaces with a reduced number of unit cells. It has been shown that a metallic parasite of the same size and located at the same distance does not provide the same performance, but rather degrades the antenna. An explanation has been given for the phenomena that make these improvements possible.

Three metasurfaces (MTS) have been designed and combined with a series end-fed 1 × 10 array antenna with a modified Dolph-Chebyshev distribution. As a result, three fully operational prototypes for imaging applications in the millimeter frequency range 24.05–24.25 GHz have been obtained, with an overall size of 86.8 × 12 × 0.762 mm^3^.

## Figures and Tables

**Table 2:** Radiation properties results obtained in measurements for the array antenna alone and combined with metasurfaces and parasite.

	Freq (GHz)	G (dBi)	SLL (dB) φ = 0°	HPBW (°) φ = 0°	SLL (dB) φ = 90°	HPBW (°) φ = 90°
**Array**	24.05	12.9				
	24.15	13.1	−13	11	─	64
	24.25	12.8				
**MTS–Array**	24.05	13.2				
	24.15	13.5	−14	11	─	59
	24.25	13.6				
**MTSsquare–Array**	24.05	10.1				
	24.15	10	−9	11	─	66
	24.25	10.1				
**MTSsquare-scaled–Array**	24.05	13.5				
	24.15	13.2	−14	12	─	58
	24.25	13.1				
**Array–Parasite**	24.05	6.3				
	24.15	5	−5	8	─	50
	24.25	4.9				

## Data Availability

The datasets generated during and/or analyzed during the current study are available from the corresponding author upon reasonable request.
